# Listening to White Noise Improved Verbal Working Memory in Children with Attention-Deficit/Hyperactivity Disorder: A Pilot Study

**DOI:** 10.3390/ijerph19127283

**Published:** 2022-06-14

**Authors:** I-Chen Chen, Hsun-Yu Chan, Keh-Chung Lin, Yu-Ting Huang, Pei-Luen Tsai, Yen-Ming Huang

**Affiliations:** 1 Department of Occupational Therapy, College of Nursing and Health Sciences, Da-Yeh University, Changhua 515006, Taiwan; 2 Department of Occupational Therapy, College of Medicine, National Cheng Kung University, Tainan 701401, Taiwan; pltsai@mail.ncku.edu.tw; 3 Department of Industrial Education, College of Technology and Engineering, National Taiwan Normal University, Taipei 106308, Taiwan; hsunyuchan@ntnu.edu.tw; 4 School of Occupational Therapy, College of Medicine, National Taiwan University, Taipei 100025, Taiwan; kehchunglin@ntu.edu.tw; 5 Department of Music, College of Human Ecology, Shih Chien University, Taipei 104336, Taiwan; yuting11@g2.usc.edu.tw; 6 Graduate Institute of Clinical Pharmacy, College of Medicine, National Taiwan University, Taipei 100025, Taiwan

**Keywords:** ADHD, working memory, arousal, white noise, moderate brain arousal model

## Abstract

Existing research demonstrates that children with attention-deficit/hyperactivity disorder (ADHD) underperform in cognitive tasks involving working memory (WM) due to hypo-arousal, which has led to the development of arousal regulation models to determine proper levels of arousal and optimal cognitive outcomes. The present study focuses on investigating the effects of external auditory stimuli on verbal WM in children with ADHD. Thirteen children with ADHD (aged 6–10 years old) and thirteen age- and gender-matched children with typical development (TD) completed the verbal WM task when listening to no sound, white noise, or pleasant music. A two-way repeated-measures analysis of variance was used to compare the verbal WM performance between groups in the three auditory conditions. Children with ADHD showed the best verbal WM performance when listening to white noise and the worst performance when listening to no sound. Yet, children with TD performed the best in the no-sound condition and the worst in the white noise condition. Our findings suggest auditory white noise is beneficial for ideal arousal regulation and cognitive performance involving verbal WM for children with ADHD and support the moderate brain arousal model. Providing external white noise is a non-invasive and cost-effective approach to improving verbal WM in children with ADHD in real-world contexts.

## 1. Introduction

Effective strategies to improve the working memory (WM) performance of children with attention-deficit/hyperactivity disorder (ADHD) are urgently needed. Characterized by a high level of inattention and hyperactivity-impulsivity [1], children with ADHD tend to have deficits in WM [2,3,4,5,6], a cognitive ability that stores and processes information and makes it available for further complex cognitive processing [7,8,9]. Such deficit is linked to a lower level of academic achievement, social skills, and organizational skills [10,11], casting a negative impact on numerous domains of development and adjustment. Although effective pharmacotherapy (e.g., methylphenidate) is available and used commonly, they often cause bothersome side effects and risks of subsequent drug overdoses or dependence [12,13]. Therefore, complementary and non-pharmacological interventions should be developed and validated to help improve the WM of children with ADHD.

One viable solution is to improve the problem of hypo-arousal, a less-than-normal level of physiological alertness [14] that is commonly observed among children with ADHD and perceived as one of the major reasons why they tend to underperform in cognitive tasks involving WM [15,16,17,18,19]. To date, scholars have proposed a number of arousal regulation models and approaches to modulate proper levels of arousal for ideal cognitive performance. For example, the optimal stimulation theory, as a homeostatic model, argues that individual regulations are needed to approach and maintain an optimal level of arousal and stimulation input. Because children with ADHD are hypo-aroused and feel unusually uncomfortable in a low-stimulation environment, the reduced stimulation of tasks may prompt stimulus-seeking and off-task behaviors, which consequently decreases task performance [20]. Similarly, according to the dopaminergic theory of positive affect, the elicited positive affect induces dopamine (DA) release, and the elevated levels of DA mediate and enhance cognitive processing [21]. Since children with ADHD have a deficiency of DA that is associated with decreased cognitive processing [18], positive affect may increase levels of DA to improve cognitive outcomes. Finally, the moderate brain arousal model claims that providing moderate external noise to individuals with low levels of DA benefits their arousal modulation and cognitive performance [19]. Given that a hypoactive DA system is positively related to a low level of neural noise, few signals passing the threshold, and attenuated cognitive processing, the cognitive performance of individuals with ADHD and a low level of tonic DA can be ameliorated after adding exogenous, moderate noise to increase neural cortical activity [19,22].

Researchers have attempted to validate the three theories, and the results indicate that particular extra stimuli may facilitate cognitive outcomes. Following the optimal stimulation theory, children with ADHD perform better on arithmetic tasks when listening to music than when listening to conversations irrelevant to the cognitive task but that are still meaningful, or when listening to no sound. However, children with typical development (TD) perform equally well under all the three auditory conditions [23]. Verifying the dopaminergic theory of positive affect, Thompson et al. (2001) found that neuro-typical adults show better performance on spatial tasks and rate higher on enjoyment scores when listening to pleasant and energic music than when listening to no sound. Yet, improved performance on spatial tasks is not found in participants who listen to sad and slow music, which is correlated with low enjoyment scores [24]. Finally, a growing body of research has joined the moderate brain arousal model suggesting that external auditory white noise is beneficial to enhancing cognitive performance. Essentially, compared with exposure to no noise, exposure to auditory white noise improves verbal memory [22], inhibition control [25], speech recognition [26], off-task behavior of children with ADHD [27], verbal memory [28,29], and the executive function of children with inattentive problems [28]. Given the usage of auditory inputs in practical settings, music and white noise have been reported to ameliorate cognitive outcomes for children with ADHD, but there is a paucity of studies that compare the effects of pleasant music and white noise on cognitive performance.

Since providing proper auditory stimuli is a feasible and cost-effective approach to improving cognitive outcomes for children with ADHD in real-world contexts, in the current study, we sought to identify what external stimulation is the most ideal and appropriate to improve cognitive performance. We compared the performance of verbal WM in the presence of three types of exogenous auditory stimuli (i.e., no sound, white noise, and pleasant music) between children with ADHD and TD to answer the following questions:

Are there differences in the performance of verbal WM between children with ADHD and TD under different auditory conditions?

Do exogenous auditory stimuli improve the performance of verbal WM for children with ADHD?

Which external auditory stimulation is the best one for children with ADHD to enhance verbal WM?

We hypothesized that children with ADHD and TD would demonstrate differences in task performance involving verbal WM in the three auditory conditions; that both white noise and pleasant music would ameliorate the performance of verbal WM for children with ADHD; and finally, that white noise would be the best auditory external input for children with ADHD for an improved verbal WM.

## 2. Materials and Methods

### 2.1. Research Design

A 2 × 3 mixed design was used in this study. The between-subjects factor was ADHD diagnosis (ADHD vs. TD), and the within-subjects factor was the auditory condition (i.e., no sound, white noise, and music). We adopted an interval of one week between each auditory condition to avoid carry-over effects. A counterbalancing procedure was applied to eliminate the order effects for repeated measures in the three conditions; the order effect was examined, and no order effect was found in this study (*F*(2, 50) = 0.28, *p* = 0.76; η^2^ = 0.01).

### 2.2. Participants

The study protocol was approved by the Institutional Review Board of National Cheng Kung University Hospital, and the participants and their parents or guardians signed the informed consent form before they began the study. We used convenience sampling to recruit participants from hospitals, clinics, and after-school programs in southern Taiwan. All participants’ verbal and non-verbal intelligence quotient (IQ) and symptoms of ADHD were assessed to ensure their eligibility for this study.

We recruited 13 boys (6 to 10 years old) diagnosed with ADHD by psychiatrists (mean age = 8.25 years) and 13 age- and gender-matched children with TD (controls; mean age = 8.42 years). One child with ADHD was taking medication but was asked to stop taking it 24 h before conducting the auditory WM tasks. Nine children in the ADHD group were undergoing rehabilitation therapy, and two children with ADHD were enrolled in special education classes at school. The TD group was recruited from after-school programs based on their scores in the Chinese version of the Swanson, Nolan, and Pelham IV (SNAP-IV) Parent Rating Scale (<95th percentile) [30]. All participants were individually evaluated for verbal and nonverbal IQs; anyone who scored below 80 was excluded from the study.

### 2.3. Measures

#### 2.3.1. Peabody Picture Vocabulary Test-Revised Edition

The Peabody Picture Vocabulary Test-Revised Edition (PPVT-R) is a norm-referenced instrument for measuring the receptive vocabulary of 3- to 12-year-old children with an established norm based on the Taiwanese population [31]. It contains 125 items with stimulus words and corresponding image plates. Each image plate includes 4 black-and-white drawings. For each item, the examiner says a word, and the examinee responds by selecting one drawing that best represents the meaning of the corresponding stimulus word. It includes two parallel forms: A and B. We used the form A. Form A has high split-half reliability (*r* = 0.90), test-retest reliability (*r* = 0.90), and concurrent validity with the Wechsler Intelligence Scale for Children-Third Edition (WISC-III; *r* = 0.69). 

#### 2.3.2. Test of Nonverbal Intelligence, Third Edition

The Test of Nonverbal Intelligence, Third Edition (TONI-3) is a norm-referenced, language-free test for measuring the aptitude, intelligence, abstract reasoning, and problem-solving abilities of 4- to 16.5-year-old children [32]. It consists of 62 items arranged in ascending order of difficulty. For each item, the examinee is presented with a set of abstract figures on the top of one page and then is required to select one best corresponding response from 4 to 6 possible responses at the bottom of that page. The test comes in two parallel forms: A and B. We used the form A, which has high internal consistency reliability (Cronbach’s α = 0.86), test-retest reliability (*r* = 0.83), and concurrent validity with Raven’s Progressive Matrices (*r* = 0.78).

#### 2.3.3. Chinese Version of the SNAP-IV Parent Rating Scale

The SNAP-IV Parent Rating Scale is a screening tool for ADHD symptoms. The norms of the parent’s scale for 6- to 13-year-old children (first to eighth grade) in Taiwan have been developed, and the 95th percentile has been recommended as the cutoff score for ADHD symptoms [30]. The scale consists of 26 items: symptoms of inattention (items 1–9), hyperactivity/impulsivity (items 10–18), and aggression/defiance (items 19–26). Parents answer the 26 items using a 4-point Likert-type scale (0 = not at all; 1 = just a little; 2 = quite a bit; 3 = very much) based on the frequency of symptom-related behaviors in their children. The scale has high internal consistency (Cronbach α = 0.90), test-retest reliability (*r* = 0.73), and concurrent validity with Conners’ Parent Rating Scales and Children Behavioral Checklist (*r* = 0.82 and 0.72, respectively).

#### 2.3.4. Digit Span Test

The Digit Span Test was pulled from the Chinese version of the Wechsler Intelligence Scale for Children, Fourth Edition to assess participants’ verbal WM. It is a standardized, norm-referenced assessment of the verbal memory of 6- to 16-year-old children with an established norm based on the Taiwanese population [33]. The examinee listens to a sequence of numerical digits at the speed of 1 digit per second and is then asked to correctly recall the digits in the same and reverse orders in forward and backward trials, respectively. The scale has high split-half reliability (*r* = 0.96), test-retest reliability (*r* = 0.94), and concurrent validity with the WISC-III (*r* = 0.58). However, according to the definition of WM [7,8,9], only the digit span backward task involves in-store and processing information, so we used the digit span backward task. An examinee who recalls the sequence correctly scores 1 point, and the highest possible score of each trial is 16. We used only one form in this study.

### 2.4. Procedure

Given the verbal tasks and different auditory conditions used in this study, we conducted the Weber test with a 512-Hz tuning fork to evaluate the hearing loss of all participants before tasks, and no participants showed hearing impairments. All participants completed the auditory WM tasks in three auditory conditions: no sound, white noise, and music (the no-sound condition was used as the control/baseline condition). The stimuli were broadcasted from two 60-Hz speakers placed 60 cm in front of the participants, whereas the auditory WM tasks were presented at 86 dB in all three auditory conditions and lasted about 15–20 min for each auditory condition.

In each condition, after a 5 min exposure to no sound, white noise, or music, the participants were asked to complete the auditory WM tasks. In the no-sound condition, there was no exogenous auditory stimulus; the average sound level was 61 dB. In the white noise condition, the white noise was presented at 78 dB. In the music condition, each participant selected one of the 10 pieces of music that they liked the most (e.g., cartoon theme song), and the chosen music was played repeatedly. The average sound level ranged from 76 to 79 dB. After a 5 min exposure to the chosen music, the participants also rated how much they liked the music selection on a 10-point Likert-type scale (0 = like the least, 10 = like the most). The 10 pieces of the music library comprised music nominated by twenty 6- to 10-year-old children who did not participate in this study as their favorite music.

### 2.5. Data Analysis

First, we used independent-samples *t*-tests, *χ*^2^ tests, and descriptive analyses to describe and compare the characteristics of participants with ADHD and TD. Second, we conducted a 3 × 2 two-way repeated-measures analysis of variance (ANOVA) with one within-subjects factor (condition: no-sound, white noise, or music) and one between-subjects factor (group: ADHD or TD) to compare the verbal WM performance of the two groups in the three auditory conditions. Given the statistically significant interaction between the condition and group (see below), we estimated the simple effects of condition and group in addition to the post hoc tests. The significance level was set at *p* < 0.05 (two-tailed), and the corresponding effect sizes were calculated. We analyzed the data with IBM SPSS Statistics for Windows, Version 26.0 (IBM Corp., Armonk, NY, USA).

## 3. Results

### 3.1. Demographics

There were no significant differences between groups in terms of age, verbal IQ, or nonverbal IQ (*t*(24) = 0.67–1.81; *p* = 0.08–0.53; *d* = 0.27–0.71). Given the relationship between IQ and cognitive performance, we included verbal IQ and nonverbal IQ as covariates in the following analyses of verbal WM. As expected, the score of inattention (*t*(24) = 4.90, *p* < 0.001; *d* = 1.92) and impulsivity (*t*(24) = 4.12, *p* < 0.001; *d* = 1.62) of SNAP-IV in the ADHD group was significantly higher than those in the TD group. There were no significant differences between groups in having experiences of learning musical instruments or habitually listening to music when doing homework (*χ*^2^ = 0.25–2.89, *df* = 1, *p* = 0.09–0.62; Table 1). Therefore, we took out music experiences as covariates in the following analyses of cognitive performance in the music condition.

### 3.2. Verbal Working Memory Performance

**Figure 1:**
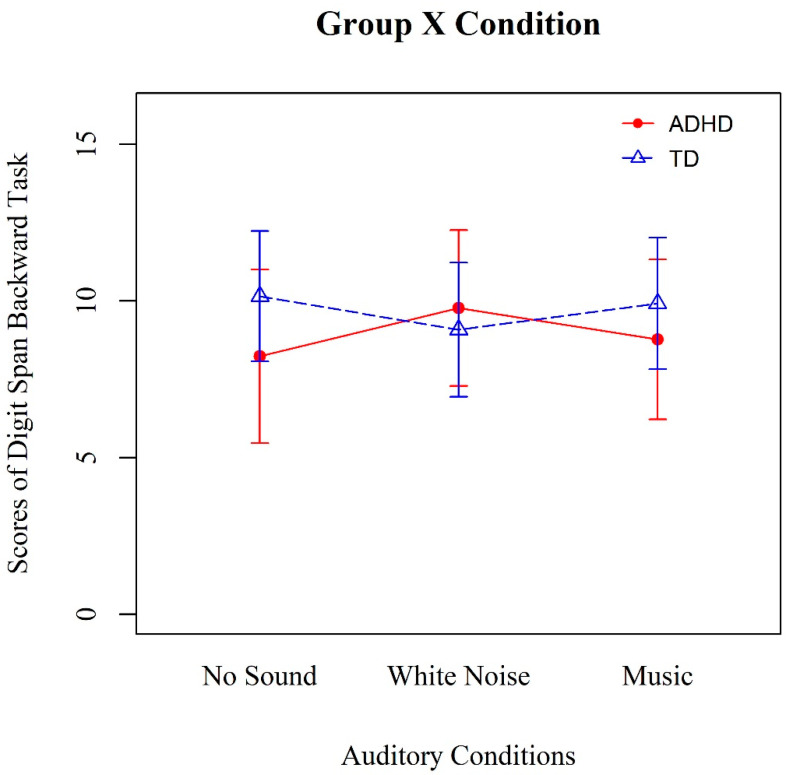
Verbal working memory performances of the two groups in the three auditory conditions.

In the repeated-measures ANOVA model, we observed a statistically significant interaction effect between group and auditory condition (*F*(2, 44) = 4.19; *p* = 0.02; η^2^ = 0.16; Figure 1). However, there were no main effects of condition (*F*(2, 44) = 0.54; *p* = 0.58; η^2^ = 0.02) and group (*F*(1, 22) = 1.02; *p* = 0.32; η^2^ = 0.04; see Table 2 for details). The ADHD group had its best memory performance when listening to white noise and its worst performance when listening to no sound; in contrast, the TD group had its best performance when listening to no sound and its worst performance when listening to white noise.

Simple effect analysis showed that, probably due to the small sample size, the ADHD group performed marginally worse in verbal WM than the TD group when listening to no sound (*F*(1, 24) = 4.01; *p* = 0.06), but there were no differences between two groups when listening to white noise (*F*(1, 24) = 0.58; *p* = 0.45) or music (*F*(1, 24) = 1.58; *p* = 0.22).

## 4. Discussion

We examined whether the auditory condition had varied effects on verbal WM performances for children with and without ADHD. Our findings suggest that adding external auditory white noise is a feasible and acceptable approach for children with ADHD to modulate arousal levels and to improve cognitive performance involving verbal WM.

Listening to exogenous white noise benefited the verbal WM of children with ADHD but listening to external pleasant music did not. Children with ADHD who have a low level of tonic DA, a low level of arousal, and a low level of neural noise may need external noise to compensate for their insufficient neural noise. The controlled exogenous white noise with steady tone and intensity may serve as additional neural noise, amplify the neural signal, and increase neural activity. As a result, this additional neural noise moderately regulates arousal and then improves the cognitive performance of children with ADHD [19,22]. However, children with TD performed worst on the verbal WM when listening to white noise. For children with TD who have a typical level of tonic DA, a typical level of arousal, and a typical level of neural noise, the external white noise may interfere with the arousal regulation and then worsen cognitive outcome [19,22]. The findings that exogenous white noise ameliorates the cognitive performance and verbal WM of children with ADHD but deteriorates that of children with TD support the moderate brain arousal model [19], and are consistent with prior studies [22,28,29]. With the simple effect analysis, there were marginal differences in no-sound (baseline) condition between the two groups, but these did not exist in the white noise condition. The effects of white noise on verbal WM in ADHD and TD groups were opposite, which narrowed the differences between the two groups. White noise has also recently been suggested as a non-pharmacological alternative for ADHD to enhance cognitive outcomes [34]. The administration of white noise through earphones is presented as a non-pharmacological treatment. It is probably free from side effects, has low cost, and does not compromise the activity of classmates; thus, it allows patients an adequate level of socialization with their peers.

The findings indicate a trend that providing external stimuli may ameliorate the verbal WM of children with ADHD but deteriorate that of children with TD. That is, adding exogenous inputs seems to benefit arousal regulation, which in turn improves cognitive outcomes for children with ADHD who are suffering from hypo-arousal, interferes with typical arousal regulation, and slows down cognitive processing for children with TD. The results suggest that children with ADHD need external stimuli from the environment to reach optimal levels of arousal and cognitive performance, which concurs with the optimal arousal theory [20].

Although exposure to pleasant music did induce positive affects in both groups (means of music enjoyment scores of 8.82 and 8.87 for the ADHD and the TD groups, respectively), the positive affect did not have beneficial impacts on the verbal WM of either group. Our findings were not aligned with the dopaminergic theory of positive affect [21] and did not coincide with previous studies [24,35]. Compared to when listening to no sound, there was a trend that the induced positive affect facilitated the verbal WM of children with ADHD when listening to pleasant music. However, the induced positive affect did not enhance the verbal WM of neuro-typical children. It is plausible that since the pleasant music consisted of meaningful phonological components (i.e., lyrics in cartoon theme songs) and frequent changes of melody, such stimuli may interfere with phonological information processing [36]. As a result, the verbal WM performance of neuro-typical children was slightly compromised and on par with that of children with ADHD.

There are some limitations to this study. As a pilot study with 26 participants, the preliminary data ought to be replicated with a larger sample size to avoid the disadvantage of low statistical power. Second, although there were neither order nor carry-over effects, we used the same verbal WM task in only one form, without parallel forms, three times for each participant. Future researchers should adopt parallel forms of the digit span task to tackle learning effect. Third, each participant possessed different levels of arousal and levels of tonic DA. However, in order to standardize the auditory conditions (e.g., presenting an identical volume of auditory stimulation), individualized levels of auditory input for each participant could not be provided in this study. To optimize children’s cognitive performance, the individualized level of white noise should be evaluated for each child when applying white noise in practical and clinical settings in the future. Finally, as our findings support the moderate brain arousal model, researchers in the future should investigate the cross-modal effect of auditory white noise on different sensory modalities and cognitive functions (e.g., changes in visuo-spatial working memory).

## 5. Conclusions

Our findings support the moderate brain arousal model that suggest that the presence of auditory white noise may enhance the moderate arousal regulation and cognitive processing of children with ADHD. When exogenous auditory white noise is provided via speakers or headphones, this non-invasive and cost-effective approach can improve the verbal WM performance of children with ADHD in daily contexts.

## Figures and Tables

**Table 1:** Characteristics of all participants.

Variables	ADHD (*n* = 13)		TD (*n* = 13)		*t*/*χ*^2^	Effect Size (Cohen’s *d*)
Age (yrs) [*M*(*SD*)]	8.25	(1.22)	8.42	(1.28)	0.36	0.27
VIQ [*M*(*SD*)]	114.54	(13.06)	106.92	(11.85)	1.56	0.71
NVIQ [*M*(*SD*)]	98.15	(15.06)	94.77	(9.23)	0.69	0.28
SNAP-IV scores						
Inattention [*M*(*SD*)]	16.46	(4.24)	7.46	(5.17)	4.85 ***	1.92
Impulsivity [*M*(*SD*)]	12.69	(5.76)	5.69	(4.63)	3.42 **	1.62
Enjoyment in music ^a^ [*M*(*SD*)]	8.82	(2.16)	8.87	(1.31)	0.07	0.54
Learn musical instrument ^b^ (*n*)	10		11		0.25	
Listen to music ^c^ (*n*)	2		6		2.89	

Note. VIQ-verbal IQ; NVIQ-nonverbal IQ; SNAP-IV-Chinese version of the Swanson, Nolan, and Pelham (Version IV); M-mean; SD-standard deviation. ^a^ participants rated how much they liked the music they listened to when doing the verbal working memory tasks on a scale of 0–10; ^b^ participants were learning musical instruments; ^c^ participants habitually listened to music when doing cognitive tasks (homework). The degree of freedom of the independent samples *t*-tests and the *χ*^2^ tests were 24 and 1, respectively. ** *p* < 0.01 *** *p* < 0.001.

**Table 2:** Two-way repeated-measures ANOVA.

Source	*df*	Mean Square	*F*	Effect Size (ŋ^2^)
Condition	2	1.74	0.54	0.02
Group	1	11.32	1.02	0.04
Condition × Group	2	13.39	4.19 *	0.16
Error	44	3.20		

Note. Verbal IQ and nonverbal IQ were included as covariates in the two-way repeated-measure ANOVA. * *p* < 0.05.

## Data Availability

The study materials and the detail of all analyses are available from the corresponding author upon reasonable request.
